# Contextual discrimination and representation in APP NL‐G‐F knockin mice

**DOI:** 10.1002/alz.091369

**Published:** 2025-01-03

**Authors:** Maya D Hopkins, Amarachi Oleru, Jayeeta Basu

**Affiliations:** ^1^ New York University Langone Health, New York, NY USA; ^2^ Neuroscience Institute, NYU Langone Health, NYU Grossman School of Medicine, New York, NY USA

## Abstract

**Background:**

The entorhinal cortex and hippocampus are loci of early vulnerability in AD. These areas are crucial for episodic memory processing for space and contexts. The majority of AD model mouse imaging and electrode studies utilize simple tasks such open field and linear track. During these paradigms, place cells in hippocampal CA1 remain functionally intact and only demonstrate perturbations later in disease progression. However, restricting behavior to these low cognitive load tasks may mask more nuanced changes in place cell activity that occur earlier in the disease. We have selected cognitively demanding tasks that recruit both episodic memory and/or odor context discrimination to reveal early‐stage deficits in task acquisition and recall.

**Method:**

In APP NL‐G‐F knock‐in homozygotes, we examined hippocampal CA1 activity through 2‐photon calcium imaging and head‐fixed behavior. Mice were aged to a “young” group (3‐5 months) or an “old” group (7‐9 months). Mice underwent two behavioral paradigms to probe episodic context discrimination. (1) A low cognitive load task where mice were exposed to new physical environments and trained to navigate to two different reward zones or (2) a high cognitive load task where mice were exposed to the same physical environment, but different contexts were established using olfactory cues and reward zones. CA1 cell bodies were imaged through viral GCaMP6f expression in excitatory cells and tracked longitudinally throughout learning. We examined the quality, flexibility, and stability of CA1 place cells in these task regimes. Finally, by targeting neuromodulatory systems that modulate GABAergic circuits, we are testing potential pharmacological rescue of functional and behavioral deficits.

**Result:**

Preliminary findings suggest an impairment in the learning rate of the high cognitive load task in homozygotes compared to wild‐type controls. Analysis is ongoing for the functional imaging of place cell activity including the stability of cells across days, the ability to remap between contexts, calcium event rate, and the proportion of reward cells.

**Conclusion:**

APP KI model mice can learn head‐fixed navigational tasks of varying cognitive loads, but learning acquisition may be impaired. We predict impairment of contextual representation within CA1 for both task regimes in the APP mice.

